# Is There a Diabetes–Kidney–Heart Continuum? Perspectives From the Results of the Cardiovascular and Renal Outcome Clinical Trials With SGLT2 Inhibitors

**DOI:** 10.3389/fcvm.2021.716083

**Published:** 2021-09-23

**Authors:** Liwen Bao, Xiufang Gao, Kun Xie, Yong Li

**Affiliations:** Department of Cardiology, Fuden University Affiliated Huashan Hospital, Shanghai, China

**Keywords:** heart failure, type 2 diabetes mellitus, renal insufficiency, CVOT, SGLT2 inhibitors

## Abstract

Heart failure is associated with a substantial risk of mortality and morbidity. Findings from recent cardiovascular outcome trials have shown promise for sodium-glucose cotransporter-2 (SGLT2) inhibitors in preventing heart failure in patients with type 2 diabetes mellitus (T2DM). Notably, the benefits of SGLT2 inhibitors were consistent despite the presence of risk factors like atherosclerosis. Increasing evidence suggests that SGLT2 inhibitors may confer their cardioprotective effects through multiple mechanisms, ranging from improving cardiac and vascular performance to metabolism. The reduction of heart failure risk by SGLT2 inhibitors may also be attributed to the preservation of renal function. Indeed, renal insufficiency is a frequent comorbidity of patients with heart failure and T2DM; hence, the natriuretic and kidney protective effects offered by SGLT2 inhibitors may contribute to limiting adverse cardiac outcomes. In this article, we discuss the latest findings from the cardiovascular and renal outcome trials, paying special attention to the interlink between heart and kidney function, and how effective treatment of heart failure—irrespective of T2DM diagnosis—may require agents that offer both cardiac and renal protection.

## Introduction

Heart failure (HF) is a fatal disease that imparts a heavy burden on patients and healthcare systems. Approximately 26 million people are affected by HF worldwide, and its prevalence is rising each year (1). Atherosclerotic cardiovascular disease (ASCVD) is considered the leading cause of HF (2). While lipid-lowering and anti-platelet aggregation therapy have reduced the risks of major adverse cardiovascular events, such progress in coronary heart disease treatment has not brought about an improvement in the morbidity and mortality risks faced by patients with HF. Data from the US National Health Interview Survey, a study that examined 677,051 adults over a period of 11.8 years, showed a significant decrease in mortality rates in major cardiovascular disease (CVD), ischemic heart disease, and stroke, but not for patients with HF and arrhythmia (3).

Diminishing renal function is one of the most potent negative catalysts for HF. A decrease in estimated glomerular filtration rate (GFR) and albuminuria may determine the risk of progression to end-stage renal disease, and identify patients with type 2 diabetes mellitus (T2DM) at markedly higher risk of HF (4). Results from recent cardiovascular outcome trials have suggested that the benefits of sodium-glucose cotransporter 2 (SGLT) inhibitors (e.g., dapagliflozin, empagliflozin and canagliflozin) derived by patients with HF can be attributed to an improvement in renal function (5–8). A systematic review and meta-analysis of these trials also uncovered that patients without ASCVD but with multiple risk factors like T2DM experienced similar cardiorenal benefits to those with ASCVD (9). Collectively, these data suggest that, besides ASCVD, other factors might be associated with the progression of HF.

We propose that the outcomes from the recent clinical trials with SGLT2 inhibitors have uncovered a link between diabetes, kidney, and heart function. This continuum may partially explain the cardiovascular and renal benefits associated with SGLT2 inhibitors in recent cardiovascular outcomes trials. Here, we provide an overview of the link between T2DM, HF and renal insufficiency, and discuss these associations in the context of cardiovascular and renal outcomes with SGLT2 inhibitors in patients with and without T2DM. We also explore the potential mechanisms underlying cardiorenal protection by SGLT2 inhibitors.

## T2DM and HF

T2DM is a well-known cause of HF independent of ASCVD (10). The underlying mechanisms include advanced glycation end-product accumulation, microvascular dysfunction, inflammation, and lipotoxicity (11). In HF cohorts, patients with T2DM experience poorer clinical outcomes than patients without T2DM (12–14). More importantly, metabolic disorders such as T2DM, rather than ASCVD, are largely responsible for the development of HF with preserved ejection fraction (HFpEF), a condition with a substantial unmet need for effective treatments (15).

## T2DM and Renal Insufficiency

T2DM is considered one of the causes of renal impairment and, consequently, HF (16, 17). During renal injury, persistent exposure to high glucose concentrations during T2DM results in increased expression and activity of SGLT2 in the proximal tubules, which in turn leads to a maladaptive increase in glucose and sodium reabsorption and decreased sodium delivery to the macula densa (18). In spite of the increased GFR, reduced exposure of the macula densa to sodium causes an afferent renal vasodilatory response and activation of the renin-angiotensin-aldosterone system (RAAS) (18, 19). Due to long-term persistent water-sodium retention, activation of these pathways contributes to the development of renal dysfunction (18, 19). Indeed, studies have shown that dysglycemia is a risk factor for the development of renal complications in patients with T2DM, as approximately 33% of patients with T2DM develop chronic kidney disease (CKD) (20). T2DM-associated CKD has now become one of the leading causes of renal failure (21).

## Renal Insufficiency and HF

Renal dysfunction itself increases the risk of HF. It has been suggested that a decline in estimated GFR is associated with higher risk for all-cause mortality and CVD (22). Cardiac and renal diseases may interact in a complex, bidirectional and interdependent manner through three central mechanisms: hemodynamic, neurohormonal, and CVD-associated mechanisms (23). Hemodynamic mechanisms include salt and water retention, which leads to fluid overload and eventually cardiac and renal venous congestion (23, 24). Neurohormonal mechanisms are mainly composed of the activation of both RAAS and the sympathetic nervous system (23). CVD-associated mechanisms comprise systemic and local inflammatory conditions that are due to the deregulated innate and adaptive immune system, bone–mineral and acid-base disorders, anemia, and cachexia (23).

## Discussion

### SGLT2 Inhibitors and Link Between T2DM, Heart and Kidney Function

There is a complex and mutually reinforcing relationship between T2DM, the kidney, and heart. These interactions appear to confer intractable changes in volume regulation, sodium homeostasis, inflammation, and metabolism that typically lead to rapid decline and early mortality. Until the emergence of SGLT2 inhibitors, breaking this vicious cycle was often difficult through traditional means.

Findings from the recent cardiovascular outcome trials have demonstrated that SGLT2 inhibitors reduced hospitalization for HF by 27–35% in patients with T2DM with or without CVD and with or without a history of HF (5–7). SGLT2 inhibitors also reduced the composite of cardiovascular death or hospitalization for heart failure by 29–31% in patients with CKD with or without T2DM, CVD or a history of HF (25, 26). In the DAPA-CKD trial, this cardiovascular benefit was consistent between diabetic (HR 0.70, 95% CI 0.53–0.92) and non-diabetic (HR 0.79, 95% CI 0.40–1.55) patients (*p*_interaction_ = 0.78) (27). The benefit of SGLT2 inhibitors in reducing the risk of HF was confirmed in the DAPA-HF and EMPEROR-Reduced trials, which evaluated the effect of dapagliflozin and empagliflozin, respectively, in patients with HF and a reduced ejection fraction irrespective of T2DM diagnosis (8, 28). In the DAPA-HF trial, over a median of 18.2 months, the risk of a composite of worsening HF (hospitalization or an urgent visit requiring intravenous therapy for HF) or cardiovascular death was reduced by 26% among patients receiving dapagliflozin when compared with the placebo group (HR 0.74, 95 CI 0.65–0.85, *p* < 0.001) (8). After a median follow-up of 16 months, empagliflozin reduced the risk of a composite of cardiovascular death or hospitalization for HF by 25% vs. placebo (HR 0.75, 95% CI 0.65–0.86, *p* < 0.001) (28). The lower rate of the composite of cardiovascular death or hospitalization for HF in the dapagliflozin vs. placebo group in the DECLARE-TIMI 58 trial (4.9 vs. 5.8%; HR 0.83, 95% CI 0.73–0.95, *p* = 0.005) was found to be due to the 27% reduction in relative risk of hospitalization for HF (HR 0.73, 95% CI 0.61–0.88) (7). It seems that SGLT2 inhibitors have their greatest and most consistent effect on reducing the relative risk of hospitalization for HF. The beneficial effect of SGLT2 inhibitors in patients with HFpEF is currently under investigation (i.e., in the EMPOROR-Preserved, PRESERVED-HF and DELIVER trials).

Meanwhile, these cardiovascular outcomes trials have also demonstrated that SGLT2 inhibitors may be renoprotective, slowing the progression of renal disease in patients with or without T2DM (7, 25–27). Notably, dapagliflozin reduced the composite of worsening of renal function, end-stage renal disease, or renal or CVD death by 39% vs. placebo in patients the DAPA-CKD trial (HR 0.61, 95% CI 0.51–0.72, *p* < 0.001) (26). This finding was consistent between diabetic (HR 0.64, 95% CI 0.52–0.79) and non-diabetic (HR 0.50, 95% CI 0.35–0.72) patients (*p*_interaction_ = 0.24) (27). However, the incidence of the pre-specified renal composite outcome in the DAPA-HF trial, defined as a decline in estimated GFR, end-stage renal disease, or renal death, was comparable between the two treatment groups (8). While promising, further evidence is required to confirm the renal benefit of SGLT2 inhibitors.

Overall, by breaking the vicious diabetes–kidney–heart cycle, the collective impact of SGLT2 inhibitors on heart and kidney function could explain why these agents may benefit all populations rather than only patients with T2DM.

### Mechanisms of Cardiorenal Protection by SGLT2 Inhibitors

**Figure 1:**
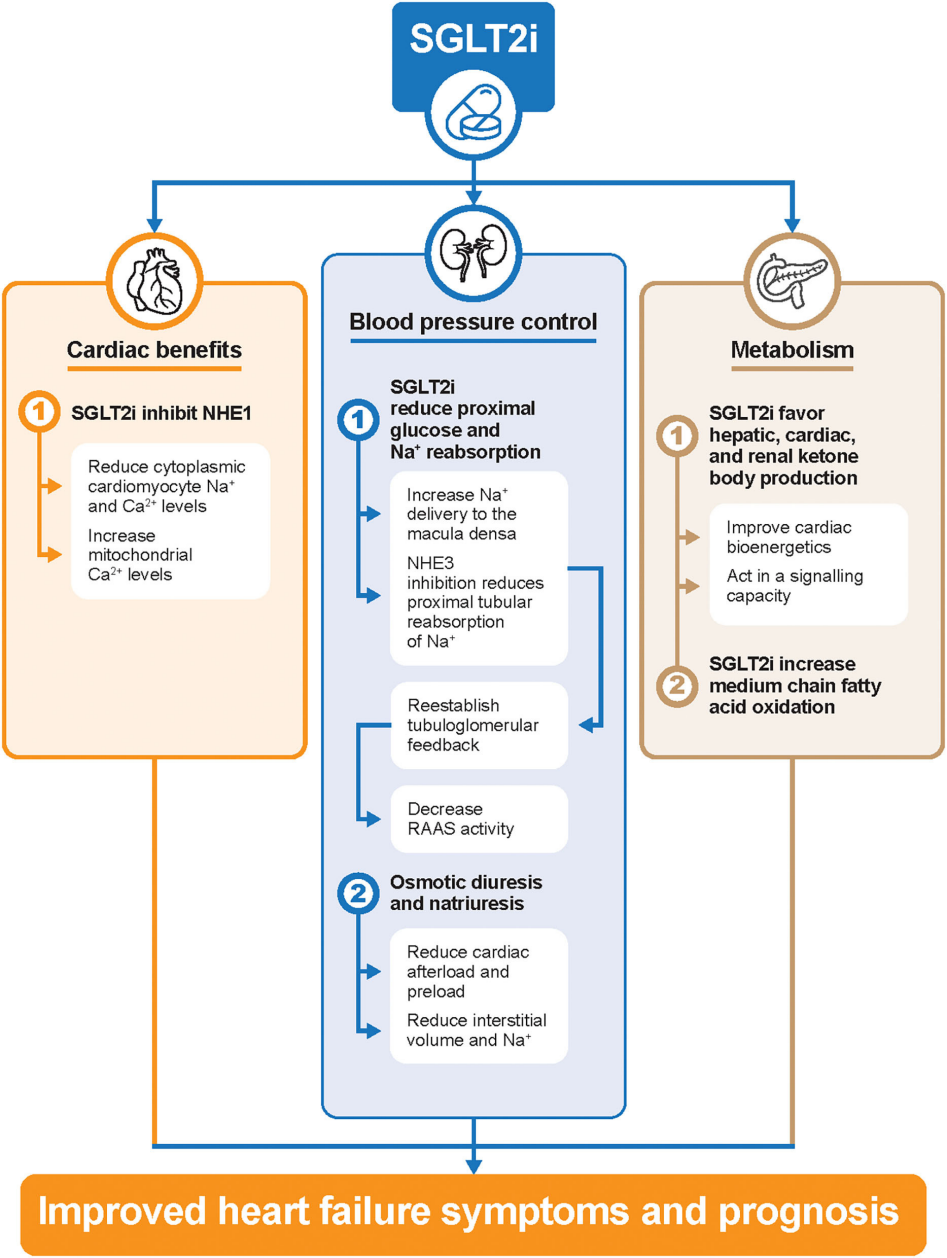
Potential cardiorenal protection mechanisms of SGLT2 inhibitors in patients with HF. Ca2+, calcium; HF, heart failure; Na+, sodium; NHE, Na+/H+ exchanger; RAAS, renin-angiotensin-aldosterone system; SGLT2i, sodium-glucose cotransporter-2 inhibitors.

Despite extensive exploratory analyses, the exact mechanisms of the salutary effects of SGLT2 inhibitors remain unclear. The reduction in glycated hemoglobin due to SGLT2 inhibitors was approximately 0.5–1.0% (29), and it seems that glucose control itself more clearly translates into a reduction of microvascular rather than macrovascular complications (30). Instead, cardiorenal protection by SGLT2 inhibitors could be explained through the following mechanisms (Figure 1). First, SGLT2 inhibitors reduce glucose and sodium reabsorption in the proximal tubule, thus increasing sodium delivery to the macula densa (31). This, in turn, restores the tubuloglomerular feedback and increases RAAS activity (31, 32). Osmotic diuresis and natriuresis can also lead to a reduction of plasma volume (33). Second, SGLT2 inhibitors may directly inhibit the Na^+^/H^+^ exchanger (NHE) 1 isoform in the myocardium, which in turn reduces cytoplasmic sodium and calcium amounts and correspondingly increases mitochondrial calcium levels (34). Natriuresis promoted by SGLT2 inhibitors has also been accounted for by the downregulation of NHE3 activity in the proximal tubules (34). Overall, the inhibition of NHE may restore whole-body sodium homeostasis and reduce cardiac failure (34). Lastly, previous data showed that SGLT2 inhibitors shift myocardial fuel metabolism away from fat oxidation toward ketone bodies (35). However, other data have shown increased short–medium chain fatty acid oxidation is responsible for cardiac metabolic remodeling, and elevated plasma ketone bodies may play additional important signaling roles in improving cardiac performances (36–38).

**Figure 2:**
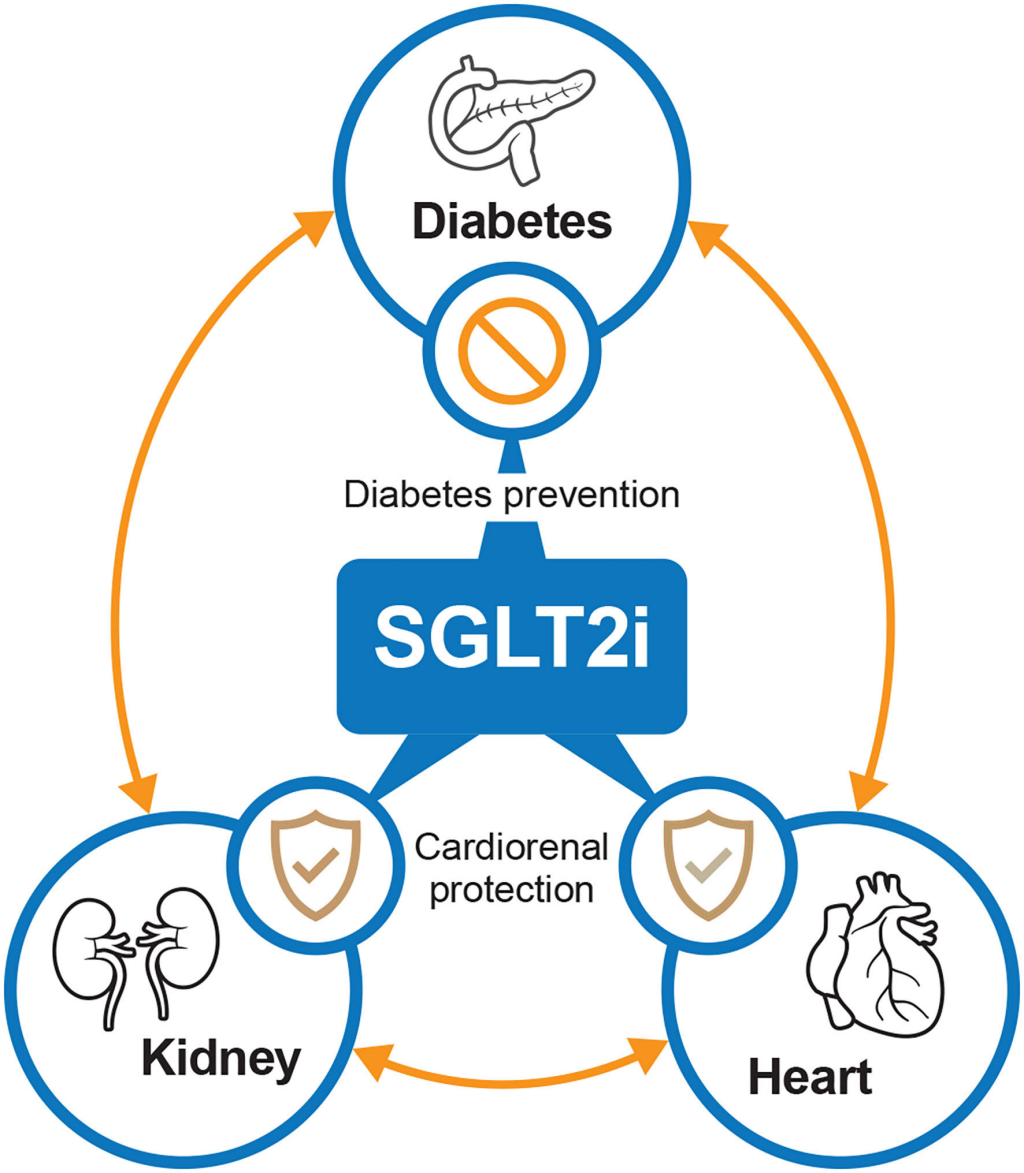
The potential interplay between SGLT2 inhibitors, diabetes, kidney, and heart. SGLT2i, sodium-glucose cotransporter-2 inhibitors.

In summary, SGLT2 inhibitors have provided rapid and significant improvements in cardio-renal outcomes (Figure 2). Specifically, demonstrable benefits in both the prevention and treatment of HF have been observed after SGLT2 inhibitor treatment. In addition, for patients with preserved renal function, SGLT2 inhibitors may represent an effective option to delay the progression of kidney disease. In the long-term, the retention of renal function can improve future cardiovascular-related outcomes by reducing the risk of both cardiovascular and all-cause death. Thus, SGLT2 inhibitors should be considered as a first-line option to decouple the diabetes–kidney–heart continuum. In other words, a new era of integrated management for HF, renal insufficiency, and T2DM is on the horizon, in which SGLT2 inhibitors will play an indispensable role.

## Author Contributions

YL contributed to the conceptualization of the article. All authors were involved in writing the original draft, all reviewed and edited the manuscript, and all read and approved the final manuscript.

## Funding

The development of the commentary and editorial assistance was funded by AstraZeneca. The sponsor had no role in the conception, design, analysis, and interpretation of this manuscript or the decision to submit the report for publication.

## Conflict of Interest

This study received funding from AstraZeneca. The funder had the following involvement with the study: funding development of the commentary and editorial assistance. The authors declare that the research was conducted in the absence of any commercial or financial relationships that could be construed as a potential conflict of interest.

## Publisher's Note

All claims expressed in this article are solely those of the authors and do not necessarily represent those of their affiliated organizations, or those of the publisher, the editors and the reviewers. Any product that may be evaluated in this article, or claim that may be made by its manufacturer, is not guaranteed or endorsed by the publisher.
